# Pembrolizumab-based systemic therapy associated with opportunities for subsequent local treatment in advanced or recurrent neuroendocrine carcinoma of the cervix: a five-case series

**DOI:** 10.1016/j.gore.2026.102156

**Published:** 2026-06-25

**Authors:** Naoyuki Ida, Shoji Nagao, Yui Tanaka, Atsushi Fujikawa, Momoko Tanioka, Ryoko Imatani, Yoshinori Tani, Hanako Sugihara, Hirofumi Matsuoka, Kazuhiro Okamoto, Junko Haraga, Hisashi Masuyama

**Affiliations:** Department of Obstetrics and Gynecology, Okayama University Graduate School of Medicine, Dentistry and Pharmaceutical Sciences, Okayama, Japan

**Keywords:** Neuroendocrine carcinoma (NEC), Cervical cancer, Pembrolizumab, Immunotherapy, Local intervention, Case series

## Abstract

**Background:**

Neuroendocrine carcinoma (NEC) of the cervix is a rare, highly aggressive malignancy with limited evidence supporting immune checkpoint blockade. We evaluated the clinical activity of pembrolizumab-based therapy in patients with advanced or recurrent cervical NEC.

**Methods:**

We retrospectively reviewed five consecutive patients with advanced or recurrent cervical NEC treated with pembrolizumab-based therapy at Okayama University Hospital between November 2022 and April 2025. Clinical characteristics, radiologic responses, molecular profiles, adverse events, local treatments, and survival outcomes were analyzed.

**Results:**

The median age was 52 years. Three patients had newly diagnosed stage IVB disease, and two had recurrent metastatic disease after radical surgery and postoperative irinotecan plus cisplatin chemotherapy. All patients received paclitaxel plus carboplatin with pembrolizumab. An objective response was observed in all patients, including one complete response and four partial responses according to RECIST version 1.1. The median maximum tumor shrinkage was 78.5%, and median progression-free survival was 10.0 months. Comprehensive genomic profiling in four patients showed microsatellite-stable tumors with low tumor mutational burden in all evaluated cases. HPV association was supported by HPV16/18 sequences in three patients and diffuse p16 expression in one additional patient. Immune-related adverse events were grade 2 or lower. In three patients, systemic disease control allowed subsequent local treatment, including radiotherapy, conversion surgery, and stereotactic radiotherapy.

**Conclusion:**

Pembrolizumab-based therapy showed encouraging clinical activity despite microsatellite-stable status and low tumor mutational burden in evaluated cases. Sustained systemic disease control may provide an opportunity for multimodal treatment incorporating subsequent local treatment.

## Highlights

• Pembrolizumab-based systemic therapy was associated with objective responses in five cervical NEC cases.
• One complete response and four partial responses were observed according to RECIST version 1.1.
• Systemic disease control allowed subsequent radiotherapy or surgery in selected patients.
• Clinical responses were observed despite microsatellite-stable tumors and low tumor mutational burden.

## 1 Introduction

Neuroendocrine carcinoma (NEC) of the cervix is a rare and highly aggressive histologic subtype, accounting for approximately 1–1.5% of all cervical malignancies (Satoh et al., 2014; Tempfer et al., 2018). It is characterized by early hematogenous dissemination, a high recurrence rate, and poor survival outcomes even in patients with apparently localized disease (Tempfer et al., 2018; Zivanovic et al., 2009). Because of its rarity, prospective clinical trials are lacking, and treatment strategies have largely been extrapolated from those established for small-cell lung cancer (Satoh et al., 2014; National Comprehensive Cancer Network, 2026).

In recent years, the addition of immune checkpoint inhibitors to platinum-based chemotherapy has significantly improved survival outcomes in extensive-stage small-cell lung cancer, establishing immunotherapy-containing regimens as a standard treatment approach (Horn et al., 2018; Paz-Ares et al., 2019). These advances have raised growing interest in applying immune checkpoint blockade to extrapulmonary neuroendocrine carcinomas, including cervical NEC. However, clinical evidence supporting the use of programmed cell death 1 (PD-1) blockade in cervical NEC remains extremely limited (Paraghamian et al., 2017; Frumovitz et al., 2020). In addition, although predictive biomarkers for immunotherapy, including PD-L1 expression, tumor mutational burden, and microsatellite instability, have demonstrated clinical relevance in other malignancies, they have not been systematically evaluated in cervical NEC because of the rarity of this disease (Paraghamian et al., 2017; Frumovitz et al., 2020; Salvo et al., 2025).

In addition to potential antitumor activity, durable systemic disease control achieved with immunotherapy-containing systemic treatment may provide an opportunity to consider subsequent local interventions, such as radiotherapy or surgery, in selected patients with metastatic disease (Palma et al., 2019). However, this treatment concept has not been sufficiently explored in cervical NEC.

Here, we report a five-case series of patients with advanced or recurrent cervical NEC treated with pembrolizumab-based systemic therapy. We aimed to evaluate clinical response, safety, molecular characteristics, and the potential role of subsequent local intervention following systemic disease control.

## 2 Patients and methods

This retrospective case series included five consecutive patients with advanced or recurrent NEC of the cervix who received pembrolizumab-based systemic therapy at the Department of Obstetrics and Gynecology, Okayama University Hospital between November 2022 and April 2025. Clinical characteristics, histopathologic findings, treatment courses, radiologic responses, genomic and immunohistochemical findings, adverse events, and survival outcomes were retrospectively reviewed using electronic medical records.

The diagnosis of cervical NEC was confirmed by experienced gynecologic pathologists through review of available pathological specimens and immunohistochemical findings. Small cell neuroendocrine carcinoma was diagnosed based on characteristic morphology, including diffuse proliferation of small tumor cells with scant cytoplasm, hyperchromatic nuclei, high mitotic activity, and apoptotic figures. Neuroendocrine differentiation was assessed using CD56, synaptophysin, chromogranin A, and/or INSM1, as available. The final diagnosis integrated morphology, immunophenotype, and clinicopathological findings, recognizing that neuroendocrine marker expression is supportive but not mandatory for diagnosis. p16 and p40 or p63 immunostaining were also reviewed when available.

Tumor responses were assessed according to Response Evaluation Criteria in Solid Tumors (RECIST), version 1.1, based on serial computed tomography or magnetic resonance imaging (Eisenhauer et al., 2009). All imaging studies were reviewed by board-certified radiologists as part of routine clinical practice. Radiologic responses were determined by the treating gynecologic oncologists based on the radiology reports and serial imaging findings. The assessments were not performed by blinded independent central review. Adverse events were graded according to the Common Terminology Criteria for Adverse Events (CTCAE), version 5.0. Comprehensive genomic profiling was performed in selected patients using FoundationOne CDx or GenMineTop, when clinically indicated. Progression-free survival was defined as the interval from initiation of pembrolizumab-based therapy to radiologic disease progression or death. Overall survival was defined as the interval from treatment initiation to death from any cause or last follow-up. The data cutoff date for survival analysis was April 30, 2026.

This study was approved by the Institutional Review Board of Okayama University Hospital (Approval No. 2311-041). Written informed consent for clinical data collection, molecular analyses, academic presentation, and publication was obtained from all patients.

## 3 Results

### 3.1 Patient characteristics

Five patients with advanced or recurrent neuroendocrine carcinoma of the cervix received pembrolizumab-based systemic therapy during the study period. Baseline clinicopathologic characteristics are summarized in Table 1.

**Table 1:** Baseline clinicopathologic characteristics of patients with cervical NEC.

A. Clinical characteristics						
**Case**	**Age**	**Setting**	**FIGO stage**	**Histology**	**Prior treatment**	**Metastatic/recurrent sites**
1	64	Newly diagnosed	IVB (cT2bN1M1)	Pure small cell NEC	None	Liver, bone, bilateral iliac LN
2	70	Newly diagnosed	IVB (cT3bN1M1)	Pure small cell NEC	None	Lung, mediastinal LN, hilar LN, gastric LN, pelvic bone
3	37	Newly diagnosed	IVB (cT3aN1M1)	Mixed NEC + HPV-associated adenocarcinoma	None	Supraclavicular LN, axillary LN, para-aortic LN, pelvic LN, bone
4	52	Recurrent	Initially IB2	Pure small cell NEC	RH + BSO + PLN + CPT-11/CDDP	Brain metastases, peritoneal dissemination
5	35	Recurrent	Initially IB1	Mixed NEC + adenosquamous carcinoma	RH + BS + PLN +CPT-11/CDDP	Vaginal stump recurrence, liver metastasis


B. Pathological and molecular characteristics					
**Case**	**IHC (NE markers)**	**Genomic profiling**	**CGP assay**	**HPV status**	**PD-L1 CPS**
1	CD56 (+), INSM1 (+)	MSS, TMB 2 muts/Mb, FBXW7 R505C	FoundationOne CDx	HPV16	Not assessed
2	Synaptophysin (+), CD56 (+)	Not performed	Not performed	Not performed	Not assessed
3	CD56 (+), Synaptophysin (+), Chromogranin A (+), INSM1 (+)	MSS, TMB 3.7 muts/Mb, KRAS G13D	GenMineTop	p16-positive; HPV type not tested	>1
4	Synaptophysin (+) Chromogranin A (+), CD56 (+)	MSS, TMB 4 muts/Mb, BCL2L1 amp ARID1A fs	FoundationOne CDx	HPV18	Not assessed
5	CD56 (+), INSM1 (+)	MSS, TMB 5 muts/Mb, PTEN loss SOX2 amp	FoundationOne CDx	HPV18	Not assessed

A. Clinical characteristics. This panel summarizes age, disease setting, FIGO stage, histologic subtype, prior treatment, and metastatic or recurrent sites in five patients with advanced or recurrent cervical neuroendocrine carcinoma.

B. Pathological and molecular characteristics. Panel B summarizes immunohistochemical findings, PD-L1 CPS status when available, comprehensive genomic profiling results, and HPV status in five patients with advanced or recurrent cervical neuroendocrine carcinoma. Comprehensive genomic profiling was performed using FoundationOne CDx or GenMineTop, as clinically indicated.

Abbreviations: BSO, bilateral salpingo-oophorectomy; BS, bilateral salpingectomy; CGP, comprehensive genomic profiling; CPT-11/CDDP, irinotecan plus cisplatin; FIGO, International Federation of Gynecology and Obstetrics; HPV, human papillomavirus; IHC, immunohistochemistry; INSM1, insulinoma-associated protein 1; LN, lymph node; MSS, microsatellite stable; NEC, neuroendocrine carcinoma; PLN, pelvic lymphadenectomy; RH, radical hysterectomy; TMB, tumor mutational burden.

The median age at treatment initiation was 52 years (range, 35–70 years). Three patients presented with newly diagnosed stage IVB disease without prior systemic therapy, and two developed recurrent metastatic disease after radical hysterectomy and postoperative irinotecan plus cisplatin chemotherapy. At recurrence, both received paclitaxel plus carboplatin with pembrolizumab. Histopathologically, three tumors were pure small cell neuroendocrine carcinoma, whereas two were mixed neuroendocrine carcinoma with HPV-associated adenocarcinoma or adenosquamous carcinoma components. Neuroendocrine differentiation was supported by immunohistochemical expression of neuroendocrine markers, including CD56, synaptophysin, chromogranin A, and/or INSM1.

Comprehensive genomic profiling was performed in four patients. In Case 2, it was not performed because only the initial biopsy specimen was available, residual tumor tissue was insufficient for additional molecular testing, and the patient did not wish to undergo further CGP testing. All evaluated tumors demonstrated microsatellite stability and low tumor mutational burden. HPV16 or HPV18 was identified in three patients, one tumor was p16-positive without HPV genotyping, and HPV testing was not performed in one patient. PD-L1 CPS was not routinely assessed at treatment initiation because pembrolizumab-based therapy was available in Japan irrespective of PD-L1 CPS status. Additional PD-L1 CPS testing using archival tumor specimens was performed only in Case 3, for whom consent for additional testing could be obtained, and showed a CPS >1.

### 3.2 Treatment response and clinical outcomes

**Fig. 1:**
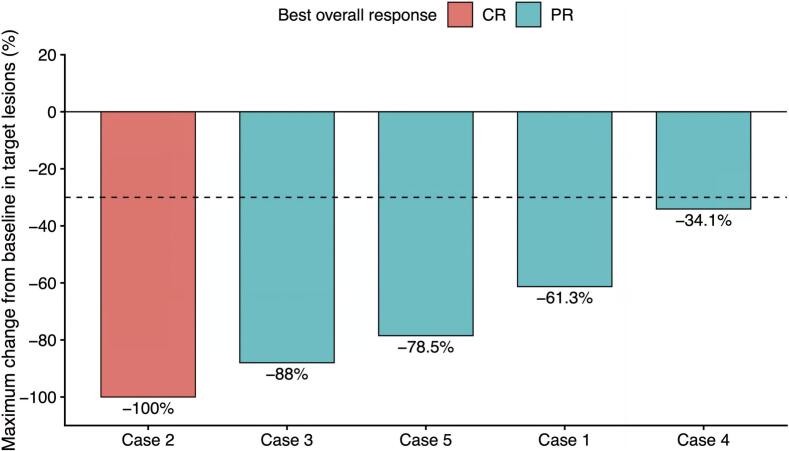
Waterfall plot of maximum tumor shrinkage during pembrolizumab-based systemic therapy.

**Fig. 2:**
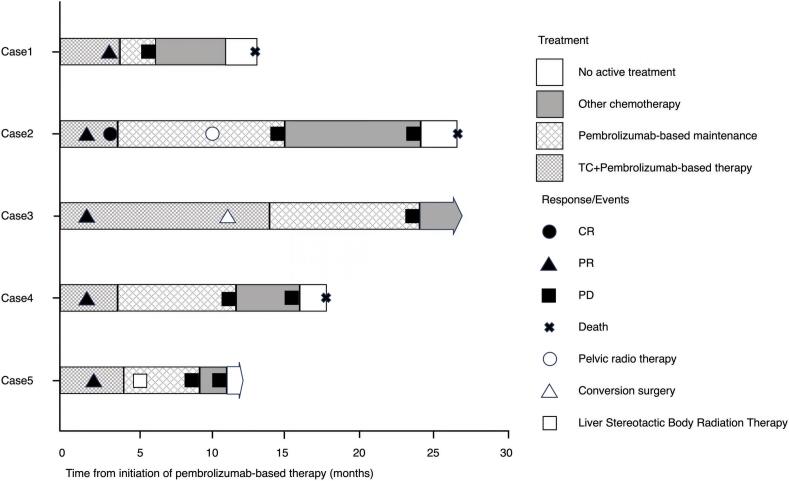
Treatment course and clinical outcomes of patients with cervical NEC treated with pembrolizumab-based therapy.

All five patients received paclitaxel plus carboplatin with pembrolizumab as systemic treatment for newly diagnosed stage IVB or recurrent metastatic disease. Treatment responses, local interventions, immune-related adverse events, and clinical outcomes are summarized in Table 2 and Fig. 1, Fig. 2. An objective response was observed in all five patients, including one complete response and four partial responses according to RECIST version 1.1. The median maximum tumor shrinkage was 78.5% (range, 34.1–100%), median time to response was 2.0 months (range, 1.2–2.5 months), median duration of response was 8.2 months (range, 5.3–21.5 months), and median progression-free survival was 10.0 months (range, 6.4–23.5 months). As shown in Table 2 and Fig. 2, subsequent treatment courses were heterogeneous. Three patients subsequently underwent local treatment after systemic disease control, as detailed below. Other patients continued pembrolizumab-based maintenance therapy or subsequently received additional systemic therapy according to disease status. The median follow-up period from the initiation of pembrolizumab-based therapy to death or last follow-up was 18.0 months (range, 12.1–27.2 months). At the data cutoff, two patients were alive, including one with ongoing disease control. Immune-related adverse events occurred in three patients, including hypothyroidism, destructive thyroiditis, and hypopituitarism. All immune-related adverse events were grade 2 or lower, and no treatment discontinuation due to immune-related toxicity was required.

**Table 2:** Treatment response, local interventions, immune-related adverse events, and outcomes.

Case	Pembrolizumab-based regimen	BOR	Maximum tumor shrinkage	TTR, months	DoR, months	PFS, months	Local intervention after response	irAE	OS, months	Status
1	TC + pembrolizumab	PR	−61.3%	1.2	5.3	6.4	None	None	13.8	Dead
2	TC + bevacizumab + pembrolizumab	CR	−100%	1.7	8.2	10.0⁎	Pelvic RT for isolated local progression	Hypothyroidism (G2)	26	Dead
3	TC + bevacizumab + pembrolizumab	PR	−88.0%	2	21.5	23.5	Surgery after response (simple hysterectomy, BSO, and pelvic lymph node biopsy)	None	27.0+	Alive
4	TC + bevacizumab + pembrolizumab	PR	−34.1%	2	10.7	12.7	None†	Destructive thyroiditis (G2) Hypopituitarism (G2)	18	Dead
5	TC + bevacizumab + pembrolizumab	PR	−78.5%	2.5	6.6	9.1	Liver SBRT	Hypothyroidism (G2)	12.1+	Alive

This table summarizes pembrolizumab-based systemic treatment regimens, best overall response, maximum tumor shrinkage, time to response, duration of response, progression-free survival, subsequent local interventions after response, immune-related adverse events, overall survival, and survival status in five patients with advanced or recurrent cervical NEC.

Abbreviations: BOR, best overall response; BSO, bilateral salpingo-oophorectomy; CR, complete response; DoR, duration of response; G, grade; irAE, immune-related adverse event; OS, overall survival; PFS, progression-free survival; PR, partial response; RT, radiotherapy; SBRT, stereotactic body radiotherapy; TC, paclitaxel plus carboplatin; TTR, time to response.

Notes: Tumor response was assessed according to RECIST version 1.1. Maximum tumor shrinkage was calculated as the greatest reduction in target lesion size from baseline. TTR, DoR, PFS, and OS were calculated from the initiation of pembrolizumab-based systemic therapy.

⁎ In Case 2, isolated local progression of the cervical primary lesion was treated with salvage pelvic radiotherapy after the initial complete response, while metastatic lesions remained controlled.

† In Case 4, whole-brain radiotherapy had been administered before pembrolizumab-based therapy and was therefore not classified as a local intervention after response.

### 3.3 Local intervention after systemic disease control

Sustained systemic disease control allowed subsequent local treatment in three patients. In Case 2, isolated local progression of the cervical primary lesion developed after an initial complete response, while metastatic lesions remained controlled. Salvage pelvic radiotherapy resulted in renewed local tumor regression and prolonged disease control.

In Case 3, marked regression of both the primary tumor and distant metastatic lesions enabled surgery after systemic therapy for initially disseminated stage IVB disease. This procedure was regarded as conversion surgery and consisted of simple hysterectomy, bilateral salpingo-oophorectomy, and pelvic lymph node biopsy. Surgical pathology demonstrated minimal residual disease consisting exclusively of NEC components.

In Case 5, substantial regression of liver metastasis during systemic therapy allowed stereotactic radiotherapy to the residual lesion, leading to further radiologic tumor shrinkage before subsequent progression.

These findings suggest that pembrolizumab-based systemic therapy may create opportunities for subsequent integrated local treatment in selected patients with cervical NEC.

## 4 Discussion

In this case series, pembrolizumab-based systemic therapy was associated with encouraging clinical activity in five patients with advanced or recurrent NEC of the cervix. An objective response was observed in all patients according to RECIST version 1.1, including one complete response and four partial responses (Eisenhauer et al., 2009). These findings are clinically notable given the historically poor prognosis of cervical NEC and the limited evidence supporting immune checkpoint blockade in this rare malignancy (Satoh et al., 2014; Tempfer et al., 2018; Zivanovic et al., 2009).

Cervical NEC is generally managed according to treatment strategies extrapolated from small-cell lung cancer because of its aggressive clinical behavior and the absence of prospective clinical trials specific to this rare malignancy (Satoh et al., 2014; Tempfer et al., 2018; Zivanovic et al., 2009; National Comprehensive Cancer Network, 2026). In recent years, multiple randomized trials in extensive-stage small-cell lung cancer have demonstrated survival benefits with the addition of immune checkpoint inhibitors to platinum-based chemotherapy (Horn et al., 2018; Paz-Ares et al., 2019), leading to the widespread adoption of immunotherapy-containing regimens. However, clinical evidence regarding PD-1 blockade in cervical NEC remains limited to small retrospective studies and isolated case reports (Paraghamian et al., 2017; Frumovitz et al., 2020). More recently, genomic studies have suggested that cervical NEC shares molecular features with HPV-associated cervical adenocarcinoma rather than classical small-cell lung cancer (Salvo et al., 2025), providing a biologic rationale for incorporating cervical cancer-based immunotherapy strategies, including pembrolizumab-containing combination therapy (Colombo et al., 2021).

In contrast, a prospective phase II study of single-agent pembrolizumab demonstrated minimal clinical activity in recurrent cervical NEC, suggesting that combination chemoimmunotherapy may be necessary to overcome intrinsic resistance in this disease (Frumovitz et al., 2020). The higher response rate observed in the present series may reflect the contribution of concurrent cytotoxic chemotherapy and, in some patients, bevacizumab; however, the small sample size and absence of a comparator group preclude any definitive conclusion regarding the relative contribution of each agent. Although etoposide plus platinum is often used for cervical NEC by extrapolation from small-cell lung cancer, no patient in this series received etoposide-containing chemotherapy. In Japan, etoposide plus platinum is not routinely approved or reimbursed for cervical NEC; therefore, treatment was selected within the approved framework for advanced or recurrent cervical cancer rather than an etoposide-based regimen. This treatment background should be considered when interpreting our findings. An important finding of the present series was that objective responses were observed despite microsatellite-stable status and low tumor mutational burden in all genomically evaluated tumors. These findings suggest that microsatellite instability and high tumor mutational burden may not be absolute prerequisites for response in cervical NEC treated with pembrolizumab-based combination therapy. Given the HPV-associated nature of cervical NEC, viral antigen-driven immunogenicity and other tumor-intrinsic or microenvironmental factors may contribute to immune responsiveness. Notably, immune-related adverse events were manageable and did not require permanent discontinuation of pembrolizumab.

A clinically relevant observation in the present series was that systemic disease control allowed subsequent local treatment in three patients. One patient underwent salvage radiotherapy for isolated local progression after complete systemic response, one underwent conversion surgery after marked tumor regression despite initially disseminated stage IVB disease, and one underwent stereotactic radiotherapy for residual liver metastasis. These cases suggest that, in selected patients, effective systemic disease control may create an opportunity for integrated local treatment as part of a multimodal strategy. This concept is consistent with emerging evidence from oligometastatic disease, including the SABR-COMET trial, suggesting that selected patients with controlled metastatic disease may benefit from local consolidative treatment strategies (Palma et al., 2019). However, whether this approach is applicable to cervical NEC requires further investigation.

Individual genomic alterations were identified in selected patients, as summarized in Table 1. Although their clinical relevance remains uncertain, these findings suggest molecular heterogeneity among cervical NEC cases (Salvo et al., 2025).

This study has several limitations. First, this was a retrospective single-institution case series with a limited sample size. Second, PD-L1 CPS was not uniformly available for all patients because PD-L1 testing was not routinely performed at treatment initiation and additional testing was limited by consent status. Third, treatment regimens were not completely uniform, including variations in bevacizumab exposure and subsequent local interventions. Nevertheless, the consistent objective responses observed across multiple patients, together with the emergence of opportunities for subsequent local intervention, support further investigation of immunotherapy-based multimodal treatment strategies in cervical NEC, consistent with the evolving concept of local consolidative treatment in controlled metastatic disease (Palma et al., 2019).

## 5 Conclusion

Pembrolizumab-based systemic therapy demonstrated encouraging clinical activity in this small series of advanced or recurrent cervical NEC, despite microsatellite-stable status and low tumor mutational burden in genomically evaluated cases. Beyond tumor regression, sustained systemic disease control allowed subsequent local treatment in selected patients, suggesting that immunotherapy-based multimodal strategies may expand therapeutic options in this aggressive disease.

## CRediT authorship contribution statement

**Naoyuki Ida:** Writing – review & editing, Writing – original draft, Visualization, Validation, Resources, Project administration, Methodology, Investigation, Formal analysis, Data curation, Conceptualization. **Shoji Nagao:** Writing – review & editing, Supervision, Project administration, Conceptualization. **Yui Tanaka:** Investigation. **Atsushi Fujikawa:** Investigation. **Momoko Tanioka:** Investigation. **Ryoko Imatani:** Investigation. **Yoshinori Tani:** Investigation. **Hanako Sugihara:** Investigation. **Hirofumi Matsuoka:** Investigation. **Kazuhiro Okamoto:** Investigation. **Junko Haraga:** Investigation. **Hisashi Masuyama:** Writing – review & editing, Supervision, Resources.

## Consent for publication

Written informed consent for publication of anonymized clinical data and radiologic/pathologic images was obtained from patients from whom consent could be obtained. For deceased patients or patients from whom direct consent could not be obtained, the requirement for individual written informed consent was waived, and an opt-out approach was used in accordance with institutional policy and the approval of the Institutional Review Board of Okayama University Hospital.

## Ethics statement

Ethical approval for this study was obtained from the Institutional Review Board of Okayama University Hospital (Approval No. 2311-041).

## Declaration of generative AI and AI-assisted technologies in the writing process

During the preparation of this work, the authors used DeepL for language editing and refinement. After using these tools, the authors reviewed and edited the content as needed and take full responsibility for the content of the publication.

## Funding

This research did not receive any specific grant from funding agencies in the public, commercial, or not-for-profit sectors.

## Declaration of competing interest

The authors declare that they have no conflicts of interest.

## Data Availability

The data that support the findings of this study are available from the corresponding author upon reasonable request.
